# Alzheimer's Disease: Tau Pathology and Dysfunction of Endocytosis

**DOI:** 10.3389/fnmol.2020.583755

**Published:** 2021-01-22

**Authors:** Kunie Ando, Sarah Houben, Mégane Homa, Marie-Ange de Fisenne, Marie-Claude Potier, Christophe Erneux, Jean-Pierre Brion, Karelle Leroy

**Affiliations:** ^1^Laboratory of Histology, Neuroanatomy and Neuropathology, Faculty of Medicine, Université Libre de Bruxelles, ULB Neuroscience Institute, Brussels, Belgium; ^2^ICM Institut du Cerveau et de la Moelle épinière, CNRS UMR7225, INSERM U1127, UPMC, Hôpital de la Pitié-Salpêtrière, Paris, France; ^3^Institut de Recherche Interdisciplinaire en Biologie Humaine et moléculaire (IRIBHM), Université Libre de Bruxelles, Brussels, Belgium

**Keywords:** Alzheimer's disease, endocytosis, genome-wide association study, phosphoinositides, tau, amyloid ß

## Introduction

Alzheimer's disease (AD) is the most common form of dementia. Its prevalence will significantly increase in the coming decades, whereas no efficient treatment is currently available. AD has two main neuropathological lesions: amyloid plaques and neurofibrillary tangles (NFTs). Amyloid plaques are composed of amyloid ß (Aß) peptides cleaved from the Amyloid Precursor Protein (APP) (Glenner and Wong, 1984). NFTs, present in the brain of AD and related neurodegenerative diseases, are constituted of tau proteins (Brion et al., 1985) in hyperphosphorylated and aggregated form (Wang and Mandelkow, 2016).

## Endolysosomal Abnormalities in AD

Evidence from both genetic and biochemical studies supports the involvement of endolysosomal abnormalities in the development of Alzheimer brain lesions (Van Acker et al., 2019). Enlargement of endosomes in neurons and peripheral cells is observed at early stage of AD and of Down syndrome (DS) individuals who are at high risk for AD (Cataldo et al., 1996; Botte and Potier, 2020). The endocytic machinery may thus be a highly vulnerable cascade that undergoes alterations at the early stages of AD. Importantly, endocytosis is closely linked to the development of both Aß and tau pathologies. On one hand, it is believed that modifications of the endolysosomal compartment in AD and DS are mostly linked to increased Aβ production as the amyloidogenic processing of APP occurs in the endosomal pathway (Koo and Squazzo, 1994; Botte et al., 2020). On the other hand, uptake of abnormal tau by endocytosis is an important step to sustain tau spreading from cell to cell (Wu et al., 2013; Evans et al., 2018; Puangmalai et al., 2020; Rauch et al., 2020). Tau pathology is strongly correlated to functional deficits in AD brains (Nelson et al., 2012). The brain propagation of tau pathology in AD follows neuroanatomical pathways and can reflect transmission of abnormal tau proteins from cell to cell in a “prion-like” manner. This transcellular transfer of abnormal tau is thought to induce recruitment and seeding of the normal soluble tau proteins into pathological aggregated tau and would need cellular internalization of abnormal tau through endocytic mechanisms (Mudher et al., 2017). In this review, we mainly focus on endocytic abnormalities in AD brains, the underlying potential mechanisms, and the relationship with tau pathology.

## Genetic risk Factors for Late-Onset AD (LOAD) and LOAD-Related Endolysosomal Proteins

Familial AD (FAD) accounts for <5% of AD cases and is well characterized by mutations in three genes (*APP, PSEN1*, and *PSEN2*). Although genetic factors are estimated to represent 60% of the risk to develop LOAD, they remained largely unknown for a long time except for *APOE* (Gatz et al., 2006). Genome-wide association studies (GWAS) and whole genome sequencing studies (WGS) have identified more than 45 genes/loci increasing or decreasing the susceptibility to develop LOAD (Lambert et al., 2013; Dourlen et al., 2019). Some GWAS hit genes encode key proteins involved in endocytosis and membrane dynamics such as INPP5D (SHIP1), Bridging Integrator 1 (BIN1), Phosphatidylinositol Binding Clathrin Assembly Protein (PICALM), Ras and Rab Interactor 3 (RIN3), CD2 Associated Protein (CD2AP), Sortilin Related Receptor 1 (SORL1), Cas scaffolding protein family member 4 (CASS4) (Lambert et al., 2013). In addition, several independent studies have reported the involvement of genes encoding endolysosomal proteins in AD such as INPPL1 (SHIP2) (Mostafavi et al., 2018), Synaptojanin-1 (SYNJ1) (Miranda et al., 2018) and phospholipase D3 (PLD3) (Cruchaga et al., 2014). The pathophysiological mechanisms by which these genes may modulate the risk for LOAD are still not fully understood.

## Dynamin-Dependent Endocytosis and LOAD-Related Endolysosomal Proteins

**Figure 1:**
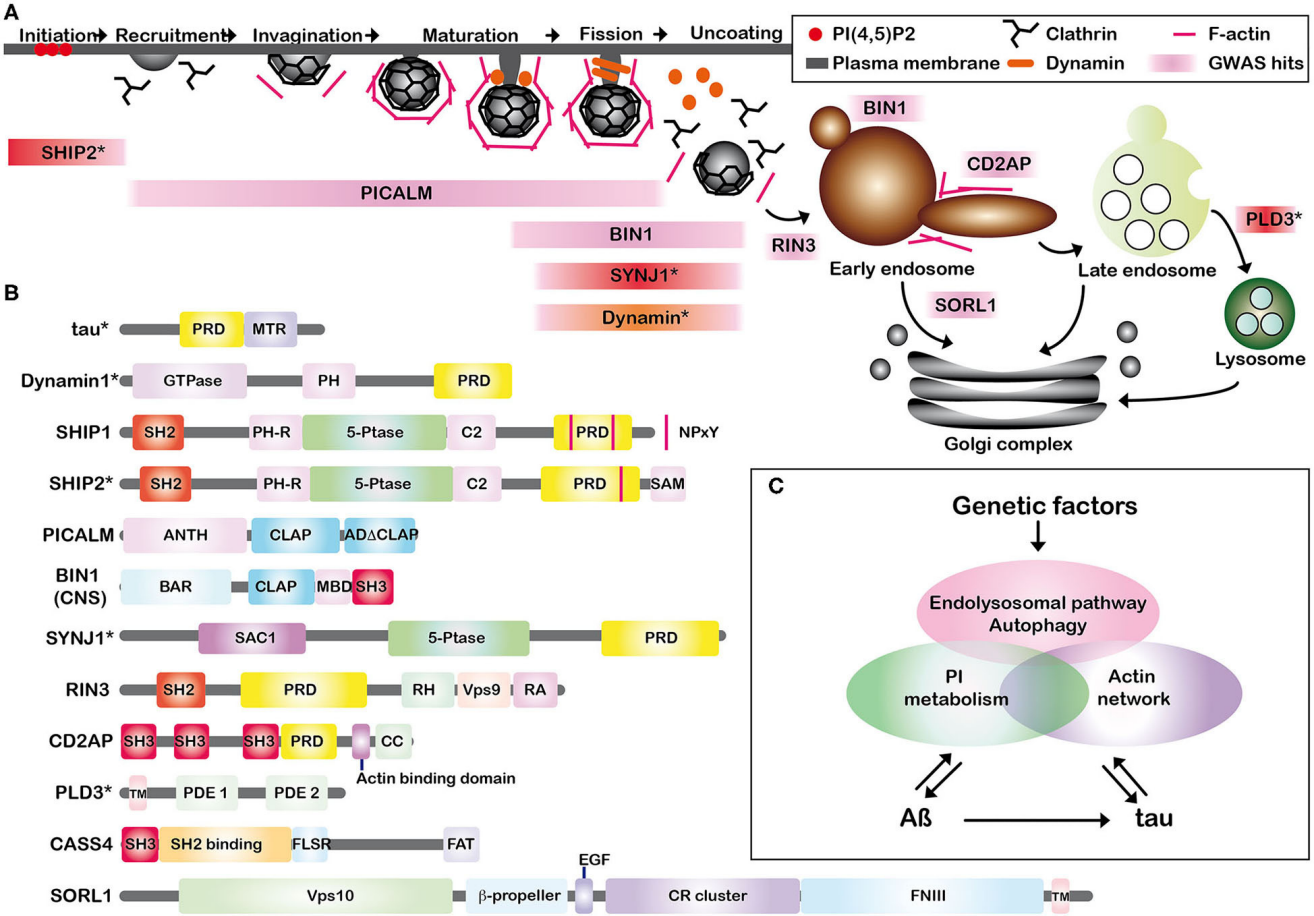
**(A)** Schematic representation of the associations of Alzheimer key proteins in CCP formation and dynamin-dependent endocytosis. Some of the recently identified AD susceptibility factors are involved in the early and late stages of dynamin-dependent endocytosis. GWAS-hits are shown in pink. SHIP1 is rather implicated in phagosome formation in macrophages and CASS4 in focal adhesion. SHIP1 and CASS4 are not included in **(A)**. *SHIP2, SYNJ1, and PLD3 are endolysosomal proteins involved in AD pathogenesis but not categorized as GWAS hits and shown in red. Dynamin is shown in orange. **(B)** The schematic illustration of the endolysosomal proteins. Some of them possess SH2, SH3 domains, or PRD. PRD, proline-rich domain; MTR, microtubule binding repeats; PH, pleckstrin-homology; SH2, Src homology 2; 5-Ptase, phosphoinositide 5-phosphatase domain; SAM, sterile alpha motif; NPxY, a conserved tyrosine phosphorylation motif (Asn-Pro-x-Tyr); ANTH, AP180 N-terminal homology (ANTH) domain; CLAP, clathrin-associated protein-binding; ADΔCLAP, A second sub-domain without clearly crucial motifs for clathrin binding. CNS, central nervous system; BAR, BIN/Amphiphysin/Rvs; SH3, Src homology 3; SAC1, suppressor of actin 1; RH, RIN-homology; Vps, vacuolar protein sorting; RA, Ras-association (RA); CC, coiled coil; TM, transmembrane; PDE, phosphodiesterase; SH2 Binding, tyrosine phosphosite-enriched domain containing binding sites for partners with SH2 domains; FLSR, central serine rich region; FAT, focal adhesion targeting domain; EGF, epidermal growth factor; CR cluster, complement-type repeat domains; FNIII, fibronectin type III repeats. *It has to be noted that tau, dynamin, SHIP2, SYNJ1, and PLD3 are not GWAS hits. **(C)** Schematic summary of our hypothesis. Genetic factors are linked to both APP processing (Aß production) and tau pathology via actin network, PI metabolism and endolysosomal pathway/autophagy, leading to neuronal dysfunctions. PI metabolism is modulated by both Aß (Berman et al., 2008) and tau (Hwang et al., 1996). Aß is linked to tau by accelerating brain tau-seeded pathology (He et al., 2018; Vergara et al., 2019).

The endolysosomal proteins listed above play critical roles at various steps of dynamin-dependent endocytosis and further steps (Figure 1A). Endocytosis starts with the recruitment of endocytic proteins to the plasma membrane subdomain enriched with phosphatidylinositol (PI) 4,5-bisphosphate [PI(4,5)P2] to form a clathrin-coated pit (CCP) (Ferguson and De Camilli, 2012; Wang et al., 2019). SHIP2 negatively regulates the dynamics of CCP formation (Nakatsu et al., 2010) by engaging a change in PI(3,4)P2 (Ghosh et al., 2018) and PI(4,5)P2 (Elong Edimo et al., 2016). Membrane invagination starts from this pit by assembling clathrin and AP-2 with the adaptor protein PICALM (Tebar et al., 1999). BIN1 is involved in membrane curvature and remodeling but BIN1 is also detected in the early endosomes of axons in neurons and is also implicated in recycling BACE1, a ß-site APP cleaving enzyme present in early endosomes (Ubelmann et al., 2017). Deep invagination of the bud and narrow neck formation are assisted by actin polymerization where SYNJ1 and dynamin interact with their protein partners possessing BAR domains (Chang-Ileto et al., 2011). After scission of newly formed vesicles by dynamin, SYNJ1 plays a critical role in clathrin-coated vesicle uncoating (Cremona et al., 1999). Then, RIN3 joins in the transport pathway from plasma membrane to early endosomes (Kajiho et al., 2003). Similarly, CD2AP is detected in the early endosomes of the dendrites in cultured neurons and is involved in actin remodeling, membrane trafficking (Lehtonen et al., 2002) and in APP sorting (Ubelmann et al., 2017). SORL1, directly interacting with APP, is localized primarily to early endosomes and the trans-Golgi network, shuttling between these two membrane compartments (Willnow et al., 2008). Lastly, PLD3 is implicated in endolysosomal system (Fazzari et al., 2017). CASS4 is rather implicated in focal adhesion integrity and tau toxicity (Dourlen et al., 2019). Most of these proteins are directly or indirectly involved in interactions with actin networks as described for dynamin (Zhang et al., 2020), SHIP1 (Lesourne et al., 2005), SHIP2 (Ghosh et al., 2018), BIN1 (Butler et al., 1997; Drager et al., 2017), SYNJ1 (Sakisaka et al., 1997), CD2AP (Lehtonen et al., 2002; Lynch et al., 2003; Tang and Brieher, 2013) and CASS4 (Deneka et al., 2015). Tau itself is also involved in organizing actin networks (Elie et al., 2015). Given that many of these endocytic proteins are also implicated in synaptic vesicle endocytosis and focal adhesion formation at the synaptic cleft, they are assumed to be involved in synaptic dysfunctions observed in AD (Dourlen et al., 2019; Perdigao et al., 2020).

## Involvement of Endocytic Proteins With Proline-Rich Domain (PRD) and Src-Homology3 (SH3)-Domain in Endocytic Alterations in AD

Dynamin-dependent endocytosis is regulated by the interplay of the interactions between PRD of dynamin and SH3 domain-containing proteins (Ferguson and De Camilli, 2012). Some of the endocytic proteins discussed above possess SH2 or SH3 domains and/or PRD (Figure 1B). These endocytic proteins form an interconnected protein network by direct or indirect protein-protein interactions. For example, BIN1 directly interacts with PICALM, SYNJ1, dynamin, RIN3 and tau (Chapuis et al., 2013; Shen et al., 2020). We hypothesize that dynamin-dependent endocytosis could be highly vulnerable and sensitive. Firstly, cyclin-dependent kinase 5 (CDK5), a kinase activated in AD brains (Patrick et al., 1999), phosphorylates PRD of both dynamin1 and SYNJ1 to block the interaction with SH3 domains of their protein partners (Ferguson and De Camilli, 2012). Secondly, the expression level of each of these endocytic proteins has a profound effect on endosomal structures. For example, depletion of SHIP2 accelerates the maturation of CCPs (Nakatsu et al., 2010) and depletion of PICALM leads to enlargement of clathrin-coated vesicle sizes (Miller et al., 2015). Likewise, depletion of BIN1, SYNJ1, CD2AP, and SORL1 results in an enlargement of early endosomes in cultured cells (Calafate et al., 2016; Ubelmann et al., 2017; Fasano et al., 2018; Knupp et al., 2020). In view of the fact that most of the LOAD-related SNPs reside in noncoding regions of the genome, they are supposed to play a role in regulating gene expression. Long-term up- or down-regulation of even one of these endocytic proteins encoded by LOAD-susceptibility genes might thus provoke endosomal abnormalities. In other words, endocytic alterations might begin much earlier than the appearance of AD lesions in the individuals bearing risk alleles of these GWAS-hit genes.

## Interactions Between Tau, Endocytosis Regulation, and Phosphoinositides

Given that endocytic proteins such as SHIP1, SHIP2, and SYNJ1 are PI-5-phosphatases involved in PI metabolism (Ramos et al., 2019), we hypothesize that upstream dysregulation of PI metabolism in AD brains may as well accelerate AD pathology. PIs act as signaling molecules in several biological functions, including membrane dynamics, cell adhesion, autophagy, and endocytosis. The homeostasis of PIs, tightly regulated by PI kinases and phosphatases in healthy cells (Di Paolo and De Camilli, 2006), is dysregulated in AD brains (Stokes and Hawthorne, 1987; Jope et al., 1994; Morel et al., 2013). While Aß modulates PI(4,5)P2 metabolism (Berman et al., 2008; He et al., 2019), PI(4,5)P2 may as well be involved in the formation of tau pathology. PI(4,5)P2 directly interacts with tau (Surridge and Burns, 1994) and can induce fibrillization of recombinant tau *in vitro* (Talaga et al., 2018). Indeed, PI(4,5)P2 is abnormally concentrated with lipid raft markers in NFTs and in granulovacuolar degeneration bodies in *post-mortem* brain tissues of AD and other tauopathies (Nishikawa et al., 2014) and is associated with several tau kinases in the raft structures (Nishikawa et al., 2016). Furthermore, tau possesses a PRD composed of seven Pro-X-X-Pro (PXXP) motifs, in its central domain (Figure 1B). Tau interacts with various SH3-containing proteins including phospholipase C (PLC) γ1 (Reynolds et al., 2008). PLC hydrolyses PI(4,5)P2 to generate diacylglycerol (DAG) and inositol 1,4,5-trisphosphate [Ins(1,4,5)P3], an important second messenger to mobilize calcium from internal stores. Tau modulates cellular signaling by interacting with PLC γ1 thereby enhancing the cleavage of PI(4,5)P2 (Hwang et al., 1996). *In vitro* phosphorylation of tau by GSK3ß, a kinase abnormally activated in AD brains (Leroy et al., 2007), significantly decreases interaction with some of its SH3-containing partners such as PLC γ1 (Reynolds et al., 2008). This implies that interactions between tau and its SH3-containing partners including PLC γ1 are likely to be disrupted in AD brains. Tau hyperphosphorylation may thus trigger dysregulation of PI metabolism and the upstream cascade of endocytosis (Wallroth and Haucke, 2018). It is also speculated that some of the proteins possessing SH2 and SH3 domains and/or PRD may be influenced by the release of “free” SH3 domains of tau partners due to tau detachment. On the other hand, the somatodendritic tau concentration is ~8-fold higher in AD compared to age-matched controls (Khatoon et al., 1992). Tau is associated with some of these endocytic proteins such as BIN1 (Calafate et al., 2016; Sartori et al., 2019), PICALM (Ando et al., 2013, 2016, 2020a) and SYNJ1 (Ando et al., 2020b). By direct interaction with tau, these endocytic proteins may (i) play roles in internalization of pathological tau during endocytosis by directly binding to tau, (ii) undergo a significant alteration in their subcellular localizations due to sequestration by tau. Some endocytic proteins interacting with phosphorylated tau are significantly decreased from the soluble fraction of AD brain lysates as observed for PICALM (Ando et al., 2016) and SYNJ1 (Ando et al., 2020b). While PICALM and SYNJ1 play critical roles in endocytosis, they also modulate autophagy (Moreau et al., 2014; Vanhauwaert et al., 2017). It is presumed that tau sequestration of such endocytic proteins could also lead to defects in both endolysosomal and autophagy pathways, central network to clearance of cellular macromolecules including Aß and tau.

## Discussion

Many of the endocytic machinery proteins implicated in AD risk possess SH2, SH3 domains, and/or PRD and are involved in actin dynamics as well as in regulation of PIs. Because the endocytic machinery needs fine-tuned regulation of PIs and endocytic protein-protein interactions, the endocytic pathway must be highly vulnerable. Dysregulation of even one of these endocytic proteins could lead to significant endocytic abnormalities. Hyperphosphorylation of tau may further accelerate endocytic dysregulation. Genetic risk factors and tau pathology might well have profound impacts on synaptic functions, endolysosomal/autophagic pathways, and APP processing via dysfunction of endocytosis, actin network, and PI metabolism (Figure 1C). Endolysosomal/autophagic abnormalities are also linked to both Aß and tau pathologies. Aß and tau are also tightly linked: Aß inhibits proteasome pathway (Almeida et al., 2006) and accelerates tau pathology progression (He et al., 2018; Vergara et al., 2019). Several genetic risk factors for LOAD may have pathological effects by inducing endocytic abnormalities leading to Aß production, tau pathology progression, synaptic failure, and deficits in membrane dynamics, all events observed in the progression of AD.

## Author Contributions

KA constructed the main concept of the manuscript by exchanging opinions with the other authors. All authors participated in writing the manuscript. All authors contributed to manuscript revision, read and approved the submitted version.

## Conflict of Interest

The authors declare that the research was conducted in the absence of any commercial or financial relationships that could be construed as a potential conflict of interest.
